# Altered lateral geniculate nucleus functional connectivity in migraine without aura: a resting-state functional MRI study

**DOI:** 10.1186/s10194-020-01086-6

**Published:** 2020-02-17

**Authors:** Di Zhang, Xiaobin Huang, Wen Su, Yuchen Chen, Peng Wang, Cunnan Mao, Zhengfei Miao, Chunmei Liu, Chenjie Xu, Xindao Yin, Xinying Wu

**Affiliations:** 1 Department of Radiology, Nanjing First Hospital, Nanjing Medical University, No.68, Changle Road, Nanjing, 210006 Jiangsu Province China; 2 Department of Neurology, Nanjing First Hospital, Nanjing Medical University, No.68, Changle Road, Nanjing, 210006 Jiangsu Province China; 3 Department of Pain Treatment, Nanjing First Hospital, Nanjing Medical University, No.68, Changle Road, Nanjing, 210006 Jiangsu Province China

**Keywords:** Migraine, Lateral geniculate nucleus, Photophobia, Functional connectivity, fMRI

## Abstract

**Objectives:**

To investigate the structural and functional connectivity changes of lateral geniculate nucleus (LGN) and their relationships with clinical characteristics in patients without aura.

**Methods:**

Conventional MRI, 3D structure images and resting state functional MRI were performed in 30 migraine patients without aura (MwoA) and 22 healthy controls (HC). The lateral geniculate nucleus volumes and the functional connectivity (FC) of bilateral lateral geniculate nucleus were computed and compared between groups.

**Results:**

The lateral geniculate nucleus volumes in patient groups did not differ from the controls. The brain regions with increased FC of the left LGN mainly located in the left cerebellum and right lingual gyrus in MwoA compared with HC. The increased FC of right LGN located in left inferior frontal gyrus in MwoA compared with HC. The correlation analysis showed a positive correlation between VLSQ-8 score and the increased FC of left cerebellum and right lingual gyrus.

**Conclusions:**

Photophobia in MwoA could be mediated by abnormal resting state functional connectivity in visual processing regions, the pain perception regulatory network and emotion regulation network. This result is valuable to further understanding about the clinical manifestation and pathogenesis of migraine.

## Introduction

Photophobia is a light-induced phenomenon that occurs in various neurological and ophthalmic diseases characterized by visual discomfort, increased headache intensity and increased brightness perception [1–4]. Over the past two decades, people have gained some scientific understanding of how specific visual pathways cause migraine photophobia in animal and human studies. One of the hotspots is the retino-thalamo-cortical pathways [2, 3, 5, 6]. From the eye to the cerebral cortex, the mechanism involve visual networks, trigeminal pain pathways and regions that regulate autonomic functions and emotions.

Photophobia during migraine attacks and interictal interval is a common feature of migraine with aura (MWA) and migraine without aura (MwoA), which can causes an aversion to light, induce or exacerbate headache [7, 8], force migraine sufferers to give up basic daily work and seek comfort in the dark. In addition, the most common aura symptom of migraine with aura is visual disturbance [8, 9]. Even among MwoA, 76% of the patients were associated with photophobia [10]. Furthermore, the pathophysiology of migraine seems a complex mechanism related to cortical spreading depression originating from the occipital lobe [11, 12], which is the center of visual cortex. Therefore, the visual network dysfunction of patients with migraine is undisputable. Some researchers have studied the presence and intensity of photosensitivity in MWA patients between episodes, using visual discomfort scores to evaluate the extent of light sensitivity [13, 14]. Structural MR studies have shown cortical visual areas changes in MwA, MwoA, or both subgroups in V2, V3A, V5 (also known as MT) [7, 11, 15–17]. Functional neuroimaging evidence over the past decade has shown specific brain function in migraine patients is abnormal compared to normal controls [10, 12, 18–22]. Some studies have shown “overactivity” of the visual cortex during migraine episodes [23] and interictal interval [24].

There are structural alteration of the visual cortical regions in migraineur with and without aura, and fMRI can show functional connection abnormalities in migraine patients. Based on the important role of the visual aura in the pathophysiology of MWA, the visual pathway has been extensively explored in MwA patients. However, there are few studies on the pathophysiology of photophobia in MwoA patients. The lateral geniculate nucleus (LGN, also known as lateral geniculate body or lateral geniculate complex) is the relay center of the visual pathway located in the posterior thalamus, just below and outside the pulvinar. It accepts the main sensory input from the retina. LGN is the major central link between the optic nerve and the occipital lobe [1]. Therefore, the purpose of this study was to investigate the structural and functional changes of LGN in MwoA, and to elucidate the mechanism of uneasy with light and light-induced attack or light enhanced attack in migraine. We hypothesized that LGN is involved in migraine visual processing and pain regulation. To address this hypothesis, we obtained structural and functional magnetic resonance images of healthy control (HC) and MwoA patients. First, the volume of LGN was automatically measured based on the structural image, and the analysis and comparison between groups was performed. Secondly, the functional connectivity of bilateral LGN was calculated using resting-state fMRI, and the changes in functional connectivity was explored between groups.

## Methods

### Participants

Between May 2018 and July 2019, a total of 52 right-handed subjects were enrolled, according to the Edinburgh Right-Handed Scale, including 30 MwoA and 22 healthy controls (HC) with similar age, gender, and education. MwoA patients were recruited from pain clinic and the Neurology Department of our hospital, and inclusion criteria were based on the International Classification of Headache Disorders, Third Edition (beta version) (ICHD − 3 beta) [8]. All patients received the Hamilton Anxiety Scale (HAMA), the Hamilton Depression Rating Scale (HAMD), the Montreal Cognitive Assessment (MOCA), the Headache Impact Test-6 (HIT-6), the Migraine Disability Assessment Questionnaire (MIDAS) and Visual Light Sensitivity Questionnaire-8 (VLSQ-8). We used the the Visual Light Sensitivity Questionnaire-8 (VLSQ-8), which contains eight-question to assess the presence and severity of visual light sensitivity [25]. Exclusion criteria are as follows: diseases affecting central nervous system function, psychotic diseases, any physical diseases such as tumors, frequent or excessive use of psychotropic substances or nuclear magnetic resonance contraindications. Healthy controls were recruited from hospital staff members and their relatives with no history of headache and family history of headache. Exclusion criteria for HC are the same as for the MwoA group. In order to avoid any possible interference of pain or pharmacological substances with fluctuations in BOLD signals, patients had no migraine and no medication for at least 3 days prior to the test and 3 days after the scan to ensure that there were no migraine during this period. In addition, in order to minimize the effects of hormones on cerebral cortex excitability, all female participants were included in the study in the middle of the cycle and excluded from pregnancy or lactation. The study was based on the recommendations and approval of the Human Research Ethics Committee of the Nanjing First Hospital. Prior to the scan, each participant received informed consent.

### Imaging methods

This study used a 3.0 T magnetic resonance imaging scanner (Ingenia, Philips Medical Systems, Netherlands) and an 8-channel head coil. All the subjects were instructed to lie in a supine position, and form padding was used to limit head movement. MRI was conducted with minimal lighting while the subject rested with eyes closed. Conventional diffuse weight images and T2-weighted images were acquired first. Whole brain three-dimensional Turbo fast echo (3D-TFE) T1WI sequence with high resolution: TR = 8.1 mm; TE = 3.7 ms; slice = 170; thickness = 1 mm; GAP = 0 mm; FA = 8 °; acquisition matrix = 256 × 256; FOV = 256 mm × 256 mm; Voxel size 1 mm*1 mm*1 mm; axially obtained functional image using gradient echo planar imaging sequence: repetition time (TR) = 2000 ms; echo time (TE) = 30 ms; number of slices = 36; thickness = 4 mm; gap = 0 mm; field of view (FOV) = 240 mm × 240 mm; acquisition matrix = 64 × 64; Voxel size 3.75 mm*3.75 mm*4 mm; flip angle (FA) = 90 °. The fMRI sequence took 8 min and 8 s.

### Image processing

**Fig. 1:**
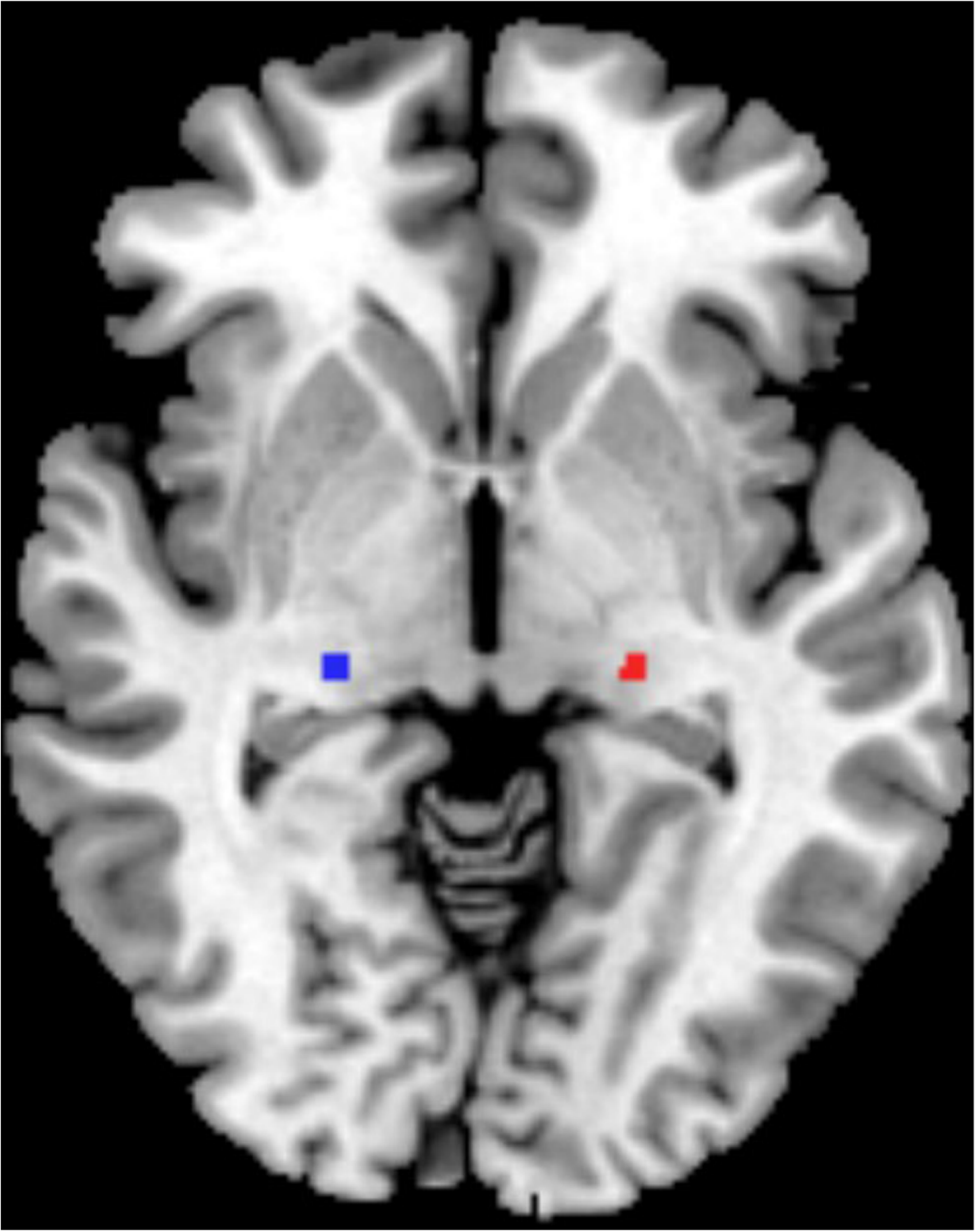
The bilateral lateral geniculate nucleus masks were generated using the WFU PickAtlas software. Red, left lateral geniculate nucleus; Blue, right lateral geniculate nucleus

Imaging data analysis was performed with the Conn toolbox3 version18b and SPM12 (www.fil.ion.ucl.ac.uk/spm/software/spm12/) running on MATLAB R2016b (MathWorks, Natick, MA). Preprocessing of 3D-TFE data includes normalized and segmented into gray matter (GM), white matter (WM), and cerebrospinal fluid (CSF) using the unified segmentation model. The GM, WM, and brain parenchyma volume were divided by the total intracranial volumes to adjust for variability due to head size. T1 images were normalized to the MNI template using affine linear registration followed by Gaussian smoothing (FWHM = 8 mm). The voxel size applied in the post-processed images was 1.5 mm*1.5 mm*1.5 mm. The seed ROI of the bilateral lateral geniculate nucleus was generated using the WFU PickAtlas software (Fig. 1). After processing volume of bilateral LGN were extracted for further statistical analysis. Preprocessing of rs-fMRI images includes rearrangement and cancellation of rotation; slice time correction; gray matter, white matter and cerebrospinal fluid segmentation; standardization of Montreal Institute of Neurology templates; and spatial smoothing based on a gaussian kernel set at 8-mm full width at half-maximum. The voxel size applied in the post-processed images was 3 mm*3 mm*3 mm. The ART-based scrubbing method, implemented in Conn, was further used to detect outlying volumes with high motion (using a 3-mm subject motion threshold and a global signal threshold set at Z = 9). Nuisance variable regression was then performed, and the first 5 principal components from the segmented white matter and CSF were regressed out of the signal. The 6 motion realignment parameters and their first-order derivatives and outlier volumes detected in the scrubbing procedure were similarly regressed out of the signal. The data were then linearly detrended, and the residual signals were bandpass filtered at 0.01 to 0.08 Hz. The data processing method was consistent with the method we used previously [26].

The seed-to-voxel method was used to detect the functional connection (FC) between the bilateral LGN and other parts of the brain. Seed-to-voxel maps were computed for each subject separately that were based on the bilateral lateral geniculate nucleus seeds. Two sample t-tests were used to study the difference in functional connectivity between the bilateral lateral geniculate nucleus of patients without aura migraine and the normal control group (using the default whole brain mask). Multiple comparison corrections were corrected by Gaussian random field (GRF), voxel level *p* < 0.01 (z > 2.58), and clustering level *p* < 0.05. The threshold for the cluster size is set to 109 voxels. To explore the relationship between functional magnetic resonance imaging (fMRI) data and clinical features, regions with significant differences between groups were extracted. Positive clusters based on RESTplus were generated as binary mask, and the connective strengths of the significant regions were extracted based on the z-maps. The average z-value of the abnormal functional junction region of each subject was then calculated.

### Statistical analysis

Demographic data differences between MwoA and HC were analyzed by between-group t-test for means and chi-square test for proportions (*p* < 0.05). Pearson correlation analysis between LGN volume, mean z values and clinical features (HAMA score, HAMD score, MoCA Score, disease duration, VAS score, monthly attack frequency, HIT-6 score, MIDAS score, VLSQ-8 score) was performed using spss 17.0 (version 17.0; spss, Chicago, Illinois, USA). Measurement data are expressed in mean and standard deviation. *P* < 0.05 was considered statistically significant and adjusted for age, sex, and years of schooling during statistical analysis in mean and correlation analysis.

## Results

### Demography, neuropsychological scores and migraine characteristics of subjects

Demography, neuropsychological scores and migraine characteristics were shown in Table 1. Age, gender, MoCA score, HAMA score, HAMD score and education years showed no significant difference between two groups. The VLSQ-8 score showed significant difference (*P* < 0.001) betweeen HC (11.50 ± 2.07) and MwoA (18.53 ± 3.81). Mean duration of disease was 9 years (range 1–20 years) and mean attack frequency was five per month (range 1–10 attacks). Thirteen (43%) of the 30 patients had usually unilateral pain, five (17%) had indeterminable side with shifting sides, twelve (40%) reported bilateral headache.

**Table 1:** Demographic and Clincal Characteristics of Participants

	MwoA patients (*n* = 30)	Healthy controls (*n* = 22)	*P* value
Age (years)	39.87 ± 10.43	34.27 ± 8.34	0.122
Gender (male/female)	4:26	8:14	0.094
MoCA score	25.87 ± 3.49	27.5 ± 1.97	0.054
HAMA score	40.2 ± 8.41	36.91 ± 5.72	0.119
HAMD score	40.68 ± 9.56	37.11 ± 6.09	0.066
Education (years)	13.57 ± 3.01	15 ± 2.49	0.075
Duration (years)	9.37 ± 7.77	NA	NA
Headache laterality, n (%)		NA	NA
unilateral	13 (43%)	NA	NA
bilateral	12 (40%)	NA	NA
shift	5 (17%)	NA	NA
Frequency (d/m)	5.17 ± 6.17	NA	NA
VAS	6.02 ± 3.11	NA	NA
Mild, n (%)	8 (27%)	NA	NA
Moderate, n (%)	17 (57%)	NA	NA
Severe, n (%)	5 (17%)	NA	NA
HIT-6 score	57.3 ± 9.27	NA	NA
MIDAS score	11.63 ± 8.76	NA	NA
VLSQ-8 score	18.53 ± 3.81	11.50 ± 2.07	<0.001

*MoCA* Montreal Cognitive Assessment, *HAMA* Hamilton anxiety scale, *HAMD* Hamilton depression scale, *HC* Healthy control, Visual Analogue Scale scale 0–10: mild1–3; moderate4–6; severe7–10.*HIT-6* Headache Impact Test-6, *MIDAS* the Migraine Disability Assessment Score, *VLSQ-8* Visual Light Sensitivity Questionnaire-8. Measurement data are expressed in mean and standard deviation

### Comparison of LGN volume between MwoA and HC

The LGN volume showed no significant difference between MwoA (left, 0.1397 ± 0.0155 ml; right, 0.1726 ± 0.0168 ml; mean, 0.1561 ± 0.0231 ml) and HC (left, 0.1455 ± 0.0110 ml; right, 0.1791 ± 0.0158 ml; mean, 0.1623 ± 0.0217 ml). The correlation analysis demonstrated that there was no significant correlation between HAMA score, HAMD score, MoCA Score, disease duration, monthly attack frequency, VAS score, HIT-6 score, MIDAS score, VLSQ-8 score and LGN volume.

### Comparison of functional connectivity of LGN between MwoA and HC

**Fig. 2:**
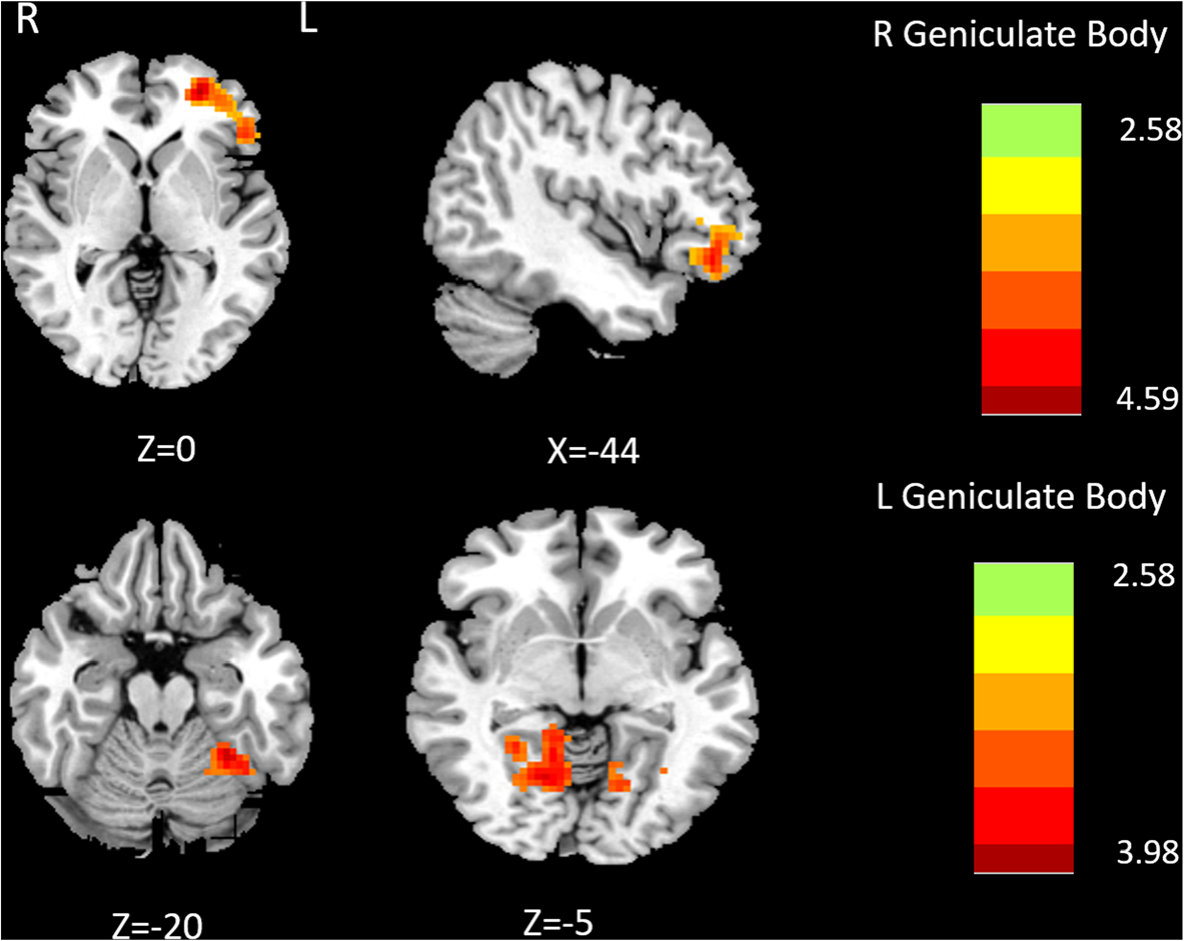
Comparison of FC of lateral geniculate nucleus between MwoA and HC. Warm color represents altered functional connectivity. MwoA patients showed increased functional connectivity compared with HC (red areas), full list of structures in Table 2. The right side of the brain refers to the left hemisphere and vice versa. The axial image was overlaid on the transverse section of the MNI-152 standard anatomical image. The z coordinate of each slice in the MNI-152 standard space is given

It was demonstrated that the brain regions with increased FC of the left LGN mainly located in the left cerebellum ([− 33–51 24], T value 3.9755) and right lingual gyrus ([15–43 -9], T value 3.9479) in MwoA compared with HC. The increased FC of right LGN located in left inferior frontal gyrus in MwoA compared with HC (Table 2 and Fig. 2). The decreased FC of bilateral LGN was not observed in MwoA compared with HC.

**Table 2:** The brain regions with Increased functional connectivity of bilateral lateral geniculate body in migrainerous without aura compared with controls

Seed	Brain region	Peak MNI coordinates			Voxel size	Peak t score
		X	Y	Z		
L_LGB	Left Cerebellum	−33	−51	−24	255	3.9755
	Right Lingual Gyrus	15	−63	−9	201	3.9479
R_LGB	Left Inferior Frontal Gyrus	−27	51	3	109	4.5855

Harvard-Oxford Cortical structural atlas

For each peak voxel x-, y-, and z-coordinates in the MNI − 152 standard space image are given

### Correlation analysis between the connection strength of positive brain regions and clinical variables

**Fig. 3:**
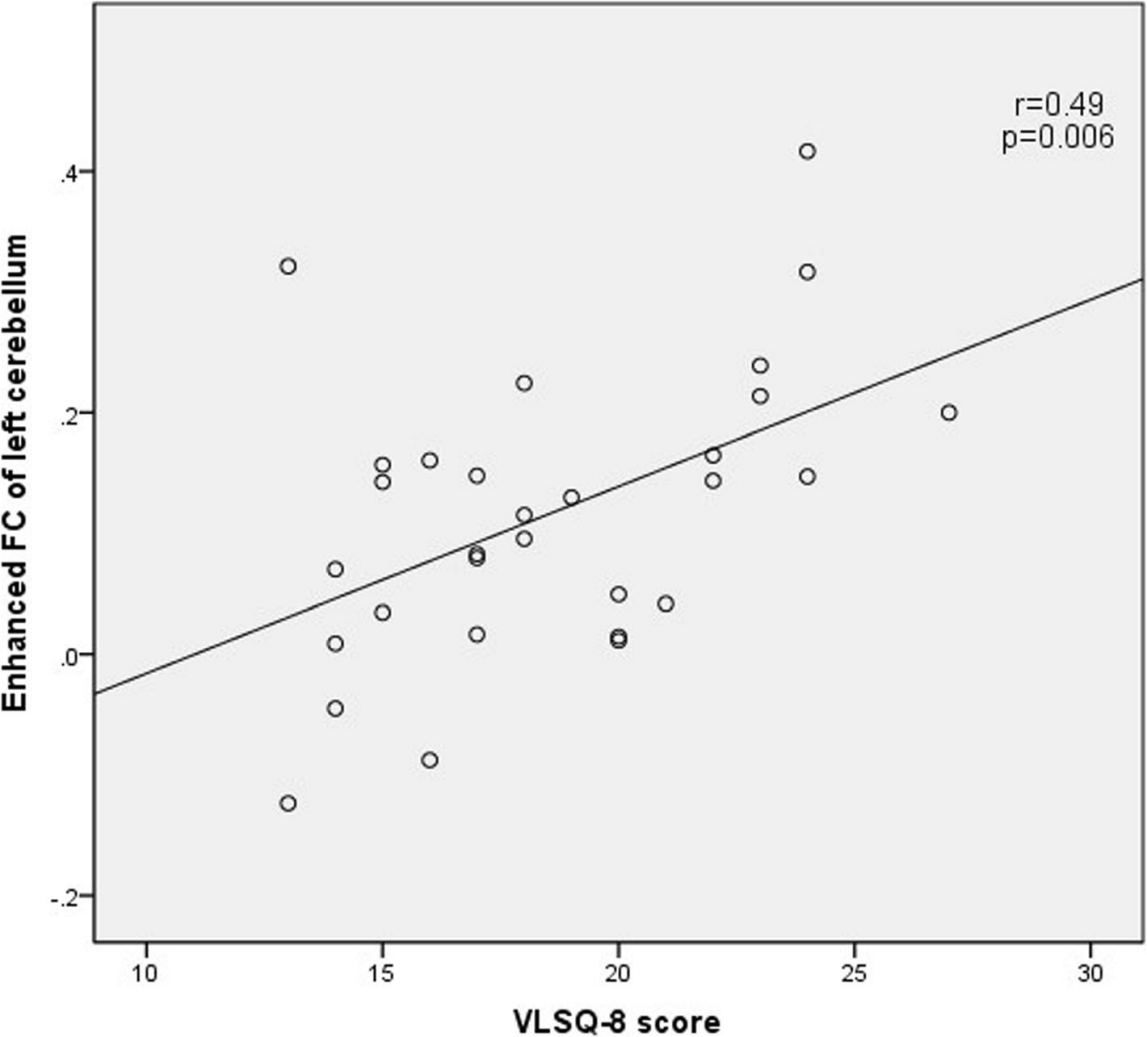
The correlation of the VLS score with the enhanced FC of left cerebellum in MwoA compared with NC

**Fig. 4:**
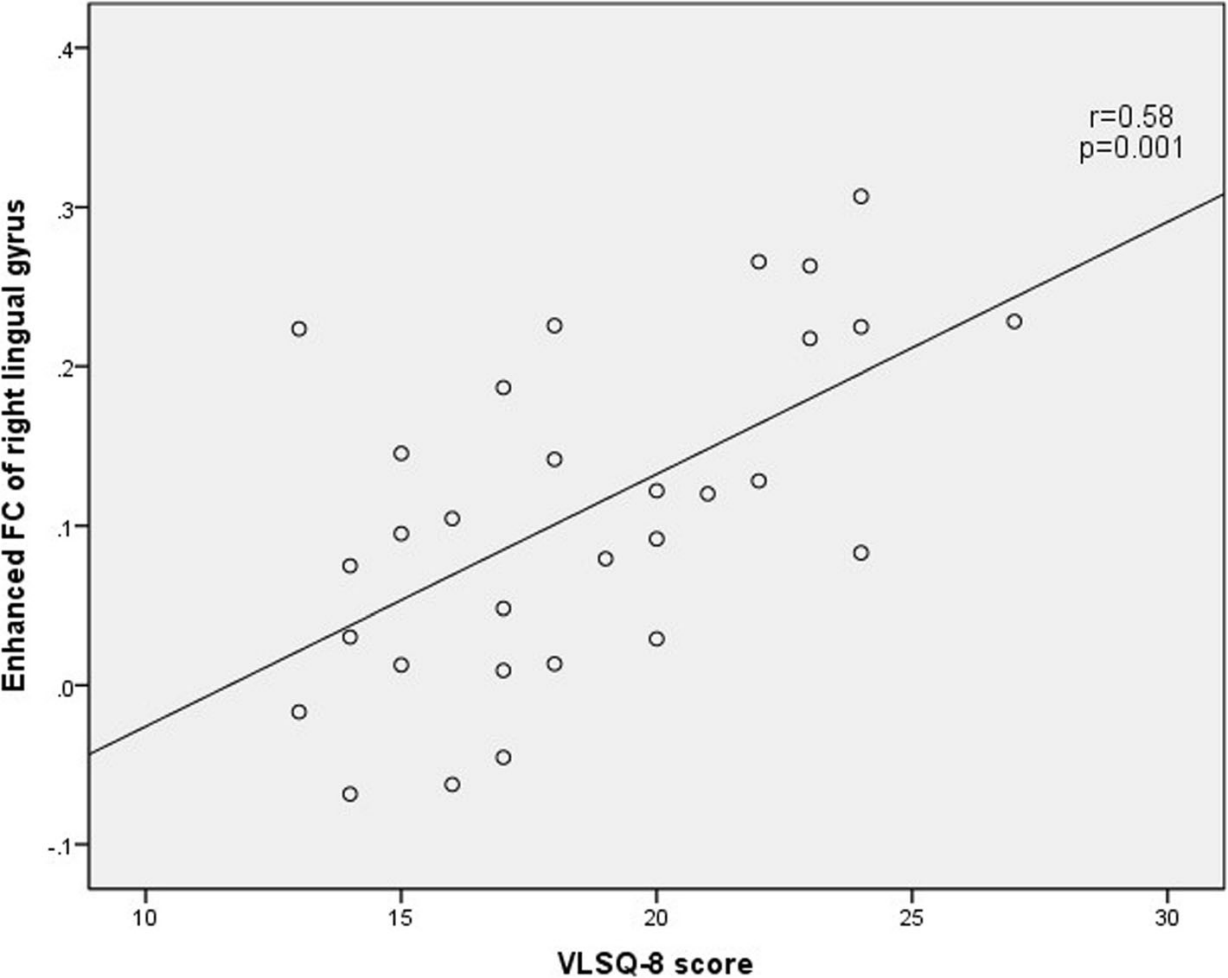
The correlation of the VLS score with the enhanced FC of right lingual gyrus in MwoA compared with NC

The correlation analysis showed a positive correlation between VLSQ-8 score and the increased FC of left cerebellum and right lingual gyrus (Fig. 3 and Fig.4). There was no statistically significant correlation between any clinical parameters of migraine severity and bold signal changes of left inferior frontal gyrus in MwoA patients (including HAMA score, HAMD score, MoCA score, disease duration, monthly attack frequency, VAS score, HIT-6 score, MIDAS score, VLSQ-8 score).

## Discussion

In migraine patients, photophobia is a common symptom, both during headache attacks and during interictal period. In the past few decades, several studies had been carried out to explore the significant role played by visual symptoms in migraineurs [11, 15, 27]. Ritobrato-Datta et al. found that MWA group had greater BOLD signal changes in V1 which responsed to visual stimuli than MwoA and control group [27]. The lateral geniculate nucleus of MWA group also responded more, but there was no significant difference between MwoA group and control group [27]. Although MwA and MwoA subjects had significantly higher levels of visual discomfort between episodes compared to control group, this was not associated with BOLD response [27]. The role of lateral geniculate nucleus in migraineurs is still unclear and controversial. Granziera C reported that the cerebral cortex in the V3A and MT regions of 24 migraineurs (12 with aura and 12 without aura) was thicker than the healthy controls, but there were no significant differences between migraineurs (MWA versus MwoA) [15]. Fractional anisotropy (FA) values in the white matter area around V3A and MT + areas on both sides of the migraine group were significantly lower than those in the HC group (except the left MT). There was no significant difference in FA values between the MWA group and the MwoA group [15]. The FA values of the superior colliculus and the left LGN in migraine patients (the two subcortical regions involved in visual motion perception) were also significantly reduced [15]. David Gaist discovered a significant statistically difference in comparison of thickness of the region of the visual regions V2 and V3A, while the thickness of the other visual regions (V1, MT) did not differ between the MWA patient and the control group [11]. On the contrary a recent study of 56 patients with migraine and a healthy control group found no cortical visual abnormalities including the somatosensory, cingulate gyrus, or V3A/MT+ [28]. It is possible that due to methodological reasons, such as sample size and software used for the measurement, these studies yielded different results. Our data show that there is no significant difference in the LGN volume between MWoA and HC, indicating that LGN volume might not suitable to be recommended as a biomarker for migraine. Correlation analysis showed that there was no significant correlation between LGN volume and neuropsychological scale score and migraine characteristics, suggesting that LGN volume possibly was not reliable to be used for migraine monitoring.

Our data showed that between MwoA and HC, right lingual gyrus have increased functional connectivity with left LGN. Compared with MwoA and healthy controls, the visual cortex connection of MwA was significantly enhanced, centered on the right lingual gyrus (LG) in a RS-fMRI study during the interictal period [29]. In addition, the surface area of the lingual cortex of MWA patients was decreased compared to HC [30]. Compared with the interictal period, during the migraine episode and light stimulation, the lingual gyrus shows hyperperfusion [24]. Recently, in a multiparametric fMRI study, MWA patients showed stronger FC in the visual network (especially the lingual gyrus) without structural or microstructural abnormalities compared with HC and MwoA patients [29]. Not only the FC of the lingual gyrus in MWA patients has changed, but also the abnormal FC of the lingual gyrus in MwoA patients has been recently discovered. Compared with HC, BOLD-response of lingual gyrus in MwoA is significantly greater during moderate painful trigeminal nerve thermal stimulation [31]. This finding increases the likelihood of “silent” lingual gyrus hyperactivity in MwoA. We can conclude that the lingual gyrus is related to visual processing and pain injury. Interesting, our results demonstrated enhanced functional connectivity between the left geniculate body and the right lingual gyrus in migraines without aura. A common belief at the structural level is that the projection fibers of the lateral geniculate body are to the ipsilateral. There is no doubt that LGN is strongly associated with ipsilateral lingual gyrus in both migraine sufferers and normal controls. Since LGN also receives signals from the contralateral optic nerve, we assume that LGN exrets a feedback effect on the contralateral visual pathway through certain pathways, thereby establishing a functional connection. This result indirectly reflects the increased excitability of the visual cortex in MwoA patients during interictal period, which may help us understand light sensitivity better in MwoA.

We observed an increase FC between left LGN and left cerebellum in MwoA compared with HC. The cerebellum have a role in nociceptive processing and in pain [32, 33]. Moulton et al. found that the cerebellar activation zone of healthy subjects overlaps with unpleasant picture viewing and heat pain and suggests that the cerebellum might have specific regions associated with general aversive processing codes and pain stimulate [33]. During nociceptive trigeminal input, ipsilateral voxel activity can be seen in cerebellar lobules VI, VIIIa and Crus I, and vermal lobule VIIIa in healthy volunteers [34]. Noxious and negative emotional picture stimuli are aversive stimuli that activate cerebellar responses [34]. Changes in experimental pain perception after cerebellar infarction also prove that cerebellum is related to pain perception [35]. Compared with the control group, patients with cerebellar infarction had significantly enhanced pain sensation to acute thermal stimulation and repeated acupuncture stimulation. Patients with thermal hyperalgesia were more pronounced on the ipsilateral side of the infarct [35]. In order to clarify the role of the cerebellum in migraine, some structural and functional studies have also been conducted. Antonio Russoa’s results showed a significant increase in cerebellar activation both in MWA and MwoA patients compared to HC patients under thermal stimulation of the trigeminal nerve [31]. The cerebellum of migraine patients (a mix group with MWA and MwoA) and controls was functionally and structurally different. In cerebellum crus, gray matter volume and neuronal activity in response to trigeminal pain increased, and its activity was regulated by migraine severity and migraine stages [36]. The cerebral cortex and subcortex have extensive connections with the cerebellum, including descending afferent and ascending efferent. The structures involved include dorsolateral prefrontal cortex, inferior parietal lobule, primary motor cortex, periaqueductal gray (PAG), parahippocampal gyrus (PHG), primary somatosensory cortex (S1), the thalamus and the hypothalamus, which are considered to be involved in sensorimotor, cognitive, pain, and emotional information processing, as well as in the pathophysiology of migraine [31, 33, 36]. Enhanced functional connectivity between left LGN and left cerebellum may demonstrated interactions between visual pathway and the pain perception regulatory network.

Furthermore, we identified increased FC between right LGN and left inferior frontal gyrus in the MWoA. Previous neuroimaging findings indicated that the inferior frontal gyrus is participated in the sensory integration and the expected reward and punishment of an action [37]. The inferior frontal gyrus cortex contribute to emotional processing in the human brain [38]. The surface area of the left inferior frontal gyrus increased significantly in the MwoA group compared to that in the normal control group [30]. Compared with HC, the BOLD response of the left inferior frontal gyrus in MwoA was significantly greater when moderately painful trigeminal nerves are thermally stimulated [31]. Some research results showed that spectrum of different colors can selectively regulate the perception of headache intensity. Green light reduces headache intensity in psychophysical research, while blue and red increases headache severity [6, 39]. Decades of research have shown that left and right frontal cortex regions are asymmetric in emotional processing. From literature, greater relative left-frontal activity has linked with approach-negative such as anger, suggesting that it is approach motivation, rather that positive or negative affect that evokes relatively greater left-frontal activity [40]. Photophobia is definitely approach-negative stimulus to migraine patients, thus it is easily to understand why there is a function connectivity between LGN and left inferior frontal gyrus. The increase of FC between right LGN and left inferior frontal gyrus may reflect the interaction between visual pathway and emotion regulation network, which may contribute to the pathophysiological mechanism of migraine patients’ aversion to light.

Correlation analysis showed that the VLSQ-8 score was positively correlated with FC increase in the left cerebellum and right lingual gyrus in MwoA compared with HC. VLS-8 was created and designed to assess the severity of VLS presence and symptoms [25]. Light sensitivity occurs both in MWA and MwoA [8]. Our results showed increased FC of right lingual gyrus and left cerebellum are associated with light sensitivity. It is difficult to determine whether light sensitivity leads to enhanced functional connectivity, or conversely, enhanced functional connectivity leads to increased photosensitivity. The next step of our study is to analyze the effective connection and derive the Granger causality connection.

Some potential limitations need to be considered. The study is cross-sectional and can therefore not discern between cause and effect. Due to the small number of MWA cases collected in our hospital, MWA patients were not included in the analysis. In addition to the laterality of the pain, the headache sites of the patients also vary greatly, so we have not analyzed the pain laterality for the time being. The potential interaction between photophobia and visual network remains inconclusive and needs to be further studied in future large sample studies.

In conclusion, although our results need to be validated using larger sample size, our results suggest that the presence of photophobia in MwoA could be mediated by abnormal resting state functional connectivity in visual processing regions, the pain perception regulatory network and emotion regulation network. Our study reveals photophobia in MwoA also has some underlying functional alterations as well as in MWA and this result is valuable to further understand the clinical manifestation and pathogenesis of migraine. Rs-fMRI finding may represent a functional biomarker for diagnosis of migraine from other types of headache patients.

## Data Availability

All data and materials generated in this study are available upon request.

## Abbreviations

CSF: Cerebrospinal fluid
FC: Functional connectivity
fMRI: Functional magnetic resonance imaging
GM: Gray matter
HAMA: The Hamilton Anxiety Scale
HAMD: The Hamilton Depression Rating Scale
HC: Healthy contral
HIT-6: The Headache Impact Test-6
LGN: Lateral geniculate nucleus
MIDAS: The Migraine Disability Assessment Questionnaire
MOCA: The Montreal Cognitive Assessment
MWA: Migraine with aura
MwoA: Migraine without aura
VAS: Visual analogue scale
VLSQ-8: Visual Light Sensitivity Questionnaire-8
WM: White matter
